# Development of a patient-derived xenograft model of glioblastoma via intravitreal injection in mice

**DOI:** 10.1038/s12276-019-0241-3

**Published:** 2019-04-16

**Authors:** Jooyoung Lee, Dong Hyun Jo, Jin Hyoung Kim, Chang Sik Cho, Jiwon Esther Han, Yona Kim, Hyoungwoo Park, Seung Ho Yoo, Young Suk Yu, Hyo Eun Moon, Hye Ran Park, Dong Gyu Kim, Jeong Hun Kim, Sun Ha Paek

**Affiliations:** 1 Department of Neurosurgery, Seoul National University Hospital, Seoul, 03080 Republic of Korea; 2 Fight against Angiogenesis-Related Blindness (FARB) Laboratory, Clinical Research Institute, Seoul National University Hospital, Seoul, 03080 Republic of Korea; 3 Tumor Microenvironment Research Center, Global Core Research Center, Seoul National University, Seoul, 08826 Republic of Korea; 4 Department of Ophthalmology, Seoul National University College of Medicine, Seoul, 03080 Republic of Korea; 5 Department of Neurosurgery, Seoul National University College of Medicine, Seoul, 03080 Republic of Korea; 6 Department of Biomedical Sciences, Seoul National University College of Medicine, Seoul, 03080 Republic of Korea; 7 Cancer Research Institute, Seoul National University College of Medicine, Seoul, 03080 Republic of Korea; 8 Hypoxia Ischemia Disease Institute, Seoul National University College of Medicine, Seoul, 03080 Republic of Korea

**Keywords:** CNS cancer, CNS cancer

## Abstract

Currently, the two primary patient-derived xenograft (PDX) models of glioblastoma are established through intracranial or subcutaneous injection. In this study, a novel PDX model of glioblastoma was developed via intravitreal injection to facilitate tumor formation in a brain-mimicking microenvironment with improved visibility and fast development. Glioblastoma cells were prepared from the primary and recurrent tumor tissues of a 39-year-old female patient. To demonstrate the feasibility of intracranial tumor formation, U-87 MG and patient-derived glioblastoma cells were injected into the brain parenchyma of Balb/c nude mice. Unlike the U-87 MG cells, the patient-derived glioblastoma cells failed to form intracranial tumors until 6 weeks after tumor cell injection. In contrast, the patient-derived cells effectively formed intraocular tumors, progressing from plaques at 2 weeks to masses at 4 weeks after intravitreal injection. The in vivo tumors exhibited the same immunopositivity for human mitochondria, GFAP, vimentin, and nestin as the original tumors in the patient. Furthermore, cells isolated from the in vivo tumors also demonstrated morphology similar to that of their parental cells and immunopositivity for the same markers. Overall, a novel PDX model of glioblastoma was established via the intravitreal injection of tumor cells. This model will be an essential tool to investigate and develop novel therapeutic alternatives for the treatment of glioblastoma.

## Brain cancer: A clearer view of glioblastoma

An improved strategy for cultivating patient-derived tumors in mice gives researchers a faster, more accurate means for testing glioblastoma treatments. Such ‘xenograft’ models are powerful tools for characterizing a patient’s cancer, but current cultivation techniques are too slow or fail to capture key features of this deadly disease. Researchers led by Jeong Hun Kim and Sun Ha Paek at Seoul National University Hospital in South Korea have demonstrated that glioblastoma cells injected into the mouse eye produce growths that mirror key characteristics of the original tumor. The tissue environment of the retina is physiologically similar to that of the brain, and cancer cells injected into the eye form glioblastoma-like tumors twice as quickly as the same cells injected into the skull. This means clinical researchers can assess drug response and accordingly adjust patient care more quickly.

## Introduction

Patient-derived xenograft (PDX) models of glioblastoma are based on the subcutaneous or intracranial injection of tumor cells into immunocompromised mice^1^. These models have been valuable tools to investigate tumor characteristics^2,3^ and the potential therapeutic efficacies of various treatment options^1,4–8^. However, via subcutaneous injection, PDX tumors develop rapidly but are confined within the subcutaneous space, which is quite different from the brain microenvironment. In contrast, through intracranial injection, tumors experience the brain microenvironment in which glioblastoma is enclosed, but tumor formation and the evaluation of therapeutic efficacy often require time. Considering that the median survival of glioblastoma patients is less than 15 months^9–11^, it is necessary to establish a PDX model of glioblastoma that can be performed more rapidly and mimic the brain microenvironment as much as possible.

In this context, it is remarkable that the retina in the eye and the brain share several neurovascular characteristics in common. First, the retina is composed of neuronal cell layers in which multiple synapses are formed among various neuronal cell types^12^. Second, microvascular endothelial cells in both the brain and the retina form blood–neural barriers with surrounding cells, including pericytes and astrocytes^13,14^. Third, other cellular components of the tumor microenvironment of glioblastoma, including microglia and immune cells, are quite similar between the brain and retina^15–17^.

In this study, a novel PDX model of glioblastoma was established through the intravitreal injection of tumor cells. Unlike U-87 MG cells, these cells from primary and recurrent glioblastoma did not form intracranial tumors. In contrast, intravitreally administered patient-derived cells formed intraocular tumors facing the retinal neuronal tissue within 4 weeks. These in vivo tumors exhibited positivity for glial fibrillary acidic protein (GFAP), vimentin, and nestin, matching the positivity of the original tumors. Furthermore, the cells isolated from in vivo tumors retained the morphological and molecular characteristics of their parental cells.

## Materials and methods

### Primary culture

Primary samples were obtained from a 39-year-old female patient and an additional four patients (demographic features are summarized in Suppl. Table 1) with glioblastoma after approval from the Institutional Review Board at Seoul National University Hospital (IRB No. H-1009–025–331). After collection during tumor resection, the samples were placed into Hank’s Balanced Salt Solution (HBSS) containing calcium and magnesium and then chopped with a surgical blade. The tissue samples were subsequently centrifuged at 1100 rpm for 4 min, rinsed with PIPES buffer, and resuspended in phosphate-buffered saline (PBS) containing trypsin-EDTA at 37 °C. Then, the tissue samples were digested with DNase I (20 U/mL) on a rocking shaker for 90 min at 37 °C, resuspended in Dulbecco’s modified Eagle’s media (DMEM) containing 10% fetal bovine serum (FBS), and centrifuged at 1100rpm for 4 min. After centrifugation, the resuspended cells were filtered through a 40-μm cell strainer and seeded in a culture flask. The cells from the primary and recurrent tumors of the 39-year-old female patient were designated GBL-28 and GBL-37, respectively. Glioblastoma cells from tumors that formed after intravitreal injection in mice were isolated using the same protocol.

### Animals

Six-week-old male Balb/c nude mice were purchased from Central Laboratory Animals and maintained under a 12-hour dark/light cycle. All animal experiments were performed in accordance with the Association for Research in Vision and Ophthalmology statement for the use of animals in ophthalmic and vision research and approved by the Institutional Animal Care and Use Committees of both Seoul National University and Seoul National University Hospital.

### Cells

U-87 MG cells (cat. no. HTB-14, ATCC) and patient-derived glioblastoma cells (GBL-15, GBL-26, GBL-28, GBL-30, GBL-37, and GBL-211) were maintained in DMEM with 10% FBS at 37 °C in a humidified atmosphere of 95% air and 5% CO_2_.

### Orthotopic transplantation of glioblastoma cells

After deep anesthesia, mice were positioned in a stereotactic frame (David Kopf Instruments). A small craniectomy was performed at 2–3 mm from the midline and 1 mm anterior to the coronal suture. Glioblastoma cells (U-87 MG, GBL-28, and GBL-37; 3 × 10^5^ cells in 5 μL) were stereotactically injected into the brain parenchyma at a depth of 3 mm. At 4–6 weeks after the injection of the tumor cells, thin sections of the mouse brain (10 μm) were processed for hematoxylin and eosin (H&E) staining and immunofluorescence staining for GFAP.

### Immunofluorescence

Thin sections of mouse brain and eyeball were washed with PBS, permeabilized with PBS containing 0.05% (v/v) saponin and 5% (v/v) normal goat serum for 3 min, and treated with PBS containing 1.5% normal goat serum for 1 h to block nonspecific binding. Then, the sections were labeled with an anti-GFAP antibody (1:100; cat. no. M0761 or Z0334, Dako), anti-human mitochondria antibody (1:100; cat. no. MAB1273, Millipore or cat. no. PA5–29550, Life Technologies), anti-vimentin antibody (1:100; cat. no. ab11256, Abcam), anti-nestin antibody (1:100; cat. no. MAB5326, Millipore), and anti-oligodendrocyte transcription factor 2 (OLIG2; 1:100; cat. no. sc-293163, Santa Cruz) overnight at 4 °C and treated with the corresponding Alexa Fluor 488- or 594-conjugated IgG antibody (1:500; cat. no. A11008, A11029, A11032, A11037, A11055, and A21207, Life Technologies) for 1 h. Nuclear staining was performed using 4’,6-diamidino-2-phenylindole dihydrochloride (DAPI, Sigma–Aldrich). Then, the slides were observed under a fluorescence microscope (Leica).

### Intravitreal injection of glioblastoma cells

Patient-derived glioblastoma cells (1 × 10^5^ cells) were injected into the vitreous cavity of 6-week-old male Balb/c nude mice. Beginning at 2 weeks after injection, eyeballs were examined daily to monitor tumor formation. A visual grading system was employed to grade the degree of tumor formation from 0–5: grade 0 (no tumor formation), grade 1 (streak-like tumor), grade 2 (plaque-like tumor), grade 3 (definite mass formation), grade 4 (vitreous-filling tumor), and grade 5 (accompanying globe enlargement or eyeball rupture), according to the standard photographs in a previous publication^18^. Two independent observers (D.H. Jo and C.S. Cho) compared the observed tumor formation with the standard photographs. There was no disagreement in the grading in this study.

### Immunocytochemistry

Glioblastoma cells were seeded in 4-well chamber slides (Nunc) and stabilized overnight. The cells were fixed with 1% paraformaldehyde at 4 °C for 10 min and permeabilized with 0.1% Triton X-100 solution (cat. no. T8787, Sigma–Aldrich) at room temperature for 3 min. After treatment with 1% bovine serum albumin to minimize nonspecific binding, the cells were labeled with an anti-GFAP antibody, anti-human mitochondria antibody, anti-vimentin antibody, and anti-nestin antibody at 4 °C overnight and treated with the corresponding Alexa Fluor 488- or 594- conjugated IgG antibody (1:500; cat.no. A11008, A11029, A11032, A11037, A11055, and A21207, Life Technologies) for 1 h. Nuclear staining was performed using DAPI. Then, the slides were observed under a fluorescence microscope (Leica).

### Statistics

Statistical analyses were performed using GraphPad Prism (GraphPad Software). A log-rank test was performed to detect statistically significant differences among the groups that underwent orthotopic transplantation of GBL-28, GBL-37, or U-87 MG cells. A *P*-value <0.05 was considered statistically significant.

## Results

### Isolation and preparation of tumor cells from a patient with primary and recurrent glioblastoma

**Fig. 1:**
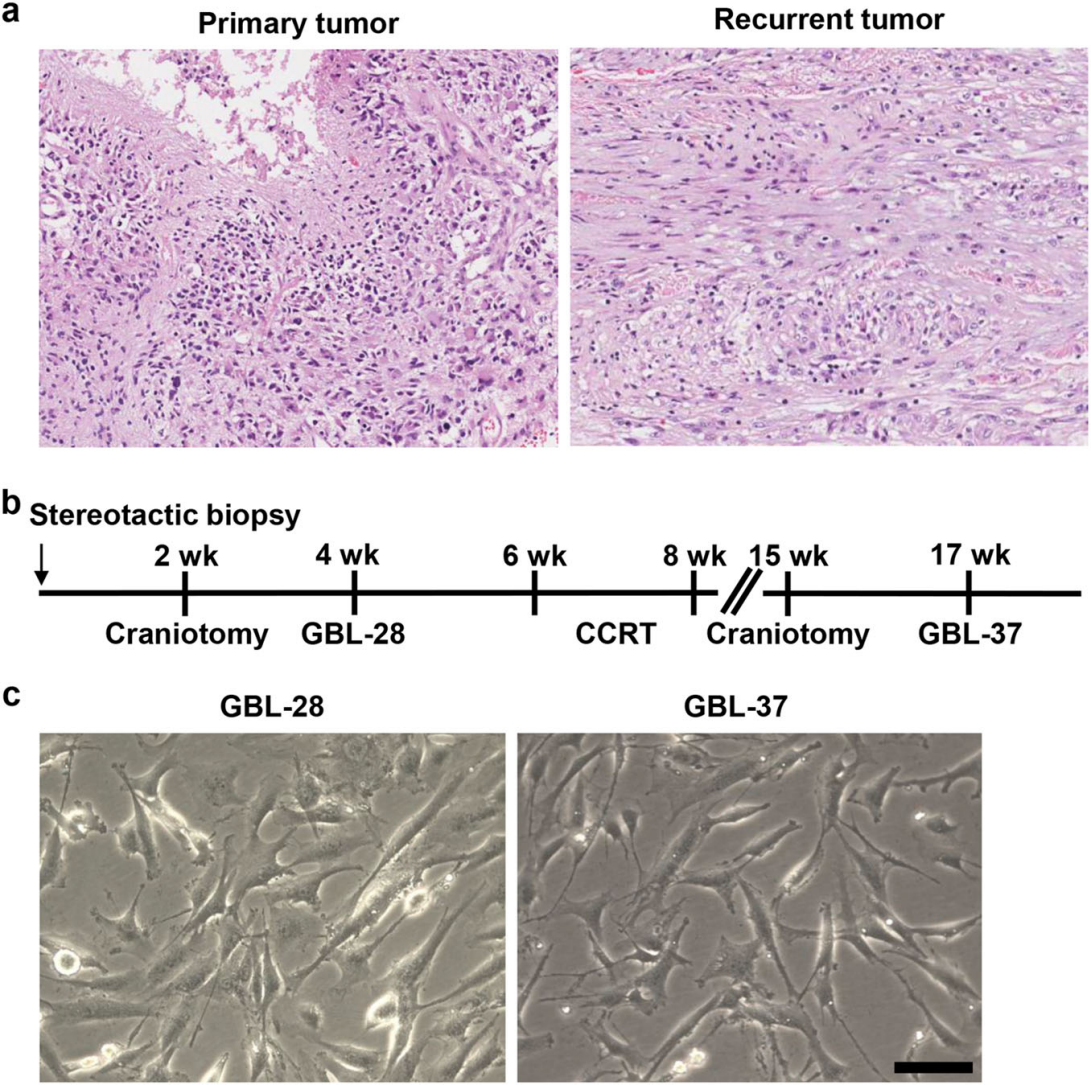
Isolation and culture of tumor cells from a patient with primary and recurrent glioblastoma. **a** Representative images of the H&E-stained sections of the primary and recurrent tumors (original magnification, 50×). **b** A schematic schedule of the stereotactic biopsy, craniotomy, concurrent chemoradiation therapy (CCRT), and preparation of the patient-derived cells (GBL-28 and GBL-37 cells). wk, week(s) after initial diagnosis and stereotactic biopsy. **c** Representative images of GBL-28 and GBL-37 cells. Scale bar: 100 μm

In a 39-year-old female patient whose initial symptom was a short-term memory defect for 2 weeks, glioblastoma was diagnosed by stereotactic brain biopsy (Fig. 1a). Then, the patient underwent surgical tumor removal and concurrent chemoradiation therapy with temozolomide. Three months after the initial surgery, another surgical tumor removal was performed to control the regrowing tumors. Histological examination revealed that both the primary and recurrent tumors were WHO grade IV glioblastoma (Table 1). Furthermore, both tumors were positive for GFAP, vimentin, and nestin by immunohistochemical analysis (Table 1). At each surgery, tumor tissue samples were prepared for primary culture, and the glioblastoma cells were designated GBL-28 (from the primary tumor) and GBL-37 (from the recurrent tumor), respectively (Fig. 1b). Both cell lines exhibited morphological characteristics of glial cells (Fig. 1c).

**Table 1:** Histological and immunohistochemical characteristics of primary and recurrent glioblastoma tumors

Characteristics	Primary tumor	Recurrent tumor
WHO grade	IV/IV	IV/IV
Increased cellularity	Present	Present
Nuclear polymorphism	Present	Present
Mitosis (pHH3)	25/10 HPF	27/10 HPF
Vascular endothelial hyperplasia	Present	Absent
Necrosis	Present	Present
Immunohistochemistry		
GFAP	Positive	Positive
Vimentin	Positive	Positive
Nestin	Positive	Positive

*HPF* high-power field, *pHH3* phosphohistone H3

### Unreliability of orthotopic transplantation of patient-derived glioblastoma cells

**Fig. 2:**
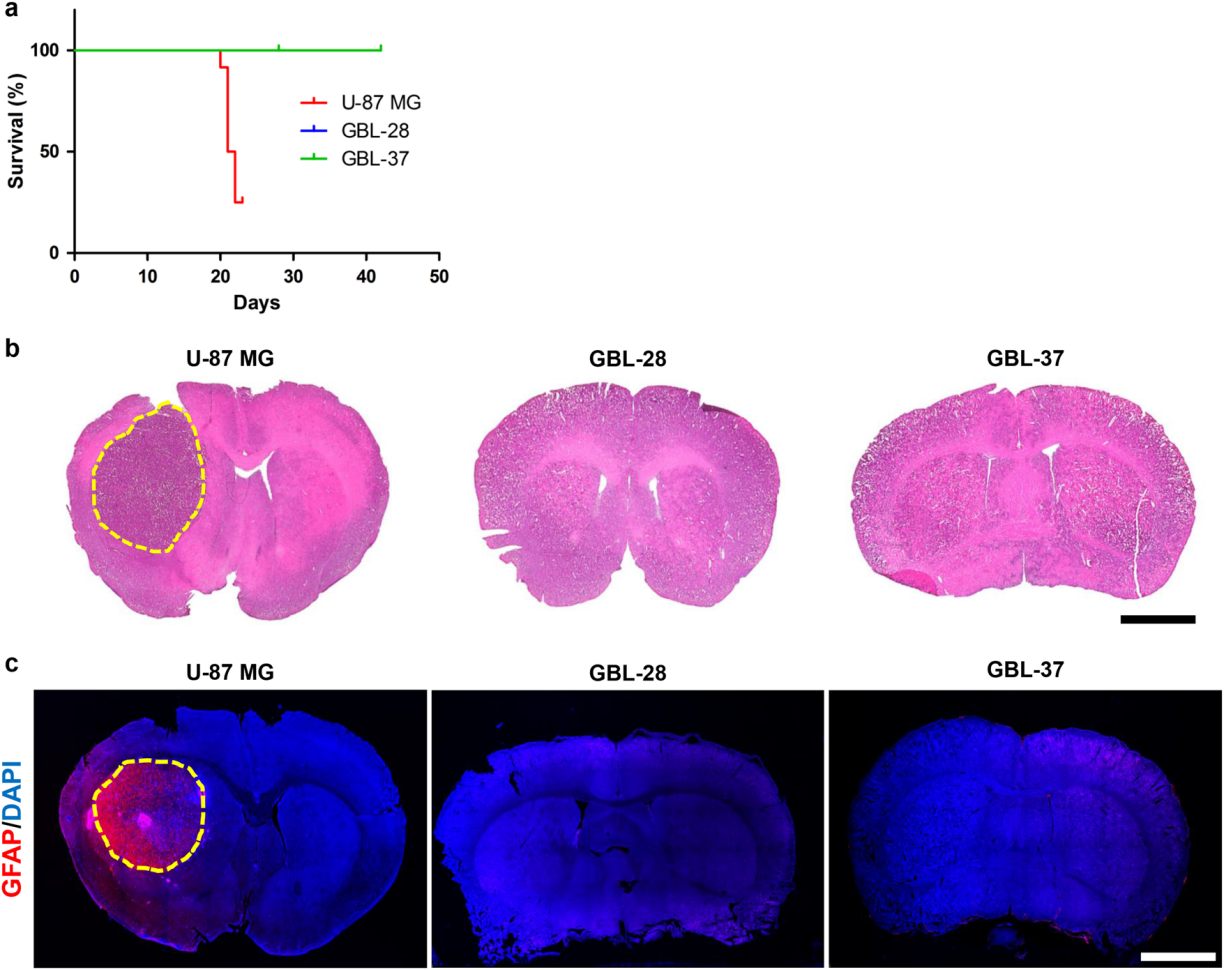
Unreliability of the orthotopic transplantation of patient-derived glioblastoma cells. **a** Kaplan–Meier survival curves of mice (*n* = 12) intracranially injected with U-87 MG, GBL-28, or GBL-37 cells. The curve for the GBL-28 group was moved upward by 2% to prevent overlap. **b** Representative images of H&E-stained sections of the brain tissue of mice at 4–6 weeks after the intracranial injection of U-87 MG (4 weeks), GBL-28 (6 weeks), or GBL-37 (6 weeks) cells. The yellow dashed lines indicate the intracranial tumors. Scale bar: 2 mm. **c** Representative images of brain sections stained using DAPI and an antibody specific for GFAP at 4–6 weeks after the intracranial injection of U-87 MG (4 weeks), GBL-28 (6 weeks), or GBL-37 (6 weeks) cells. The yellow dashed lines indicate the intracranial tumors. Scale bar: 2 mm

Although orthotopic transplantation has been repeatedly performed by researchers, including our group^2,19–22^, some patient-derived cells formed intracranial tumors after more than 6 weeks or failed to develop tumors. When 3 × 10^5^ U-87 MG, GBL-28, or GBL-37 cells were injected into the striatum of Balb/c nude mice, the survival patterns were quite different between the group injected with U-87 MG cells and the groups injected with the patient-derived glioblastoma cells (*P*-value < 0.0001; Fig. 2a). Histological and immunohistochemical examinations demonstrated that the U-87 MG cells formed well-demarcated intracranial tumors (Fig. 2b, c, left), while the GBL-28 and GBL-37 cells did not establish any tumors, even at 6 weeks after intracranial injection (Fig. 2b, c, middle and right).

### Development of a novel PDX model of glioblastoma via intravitreal injection

**Fig. 3:**
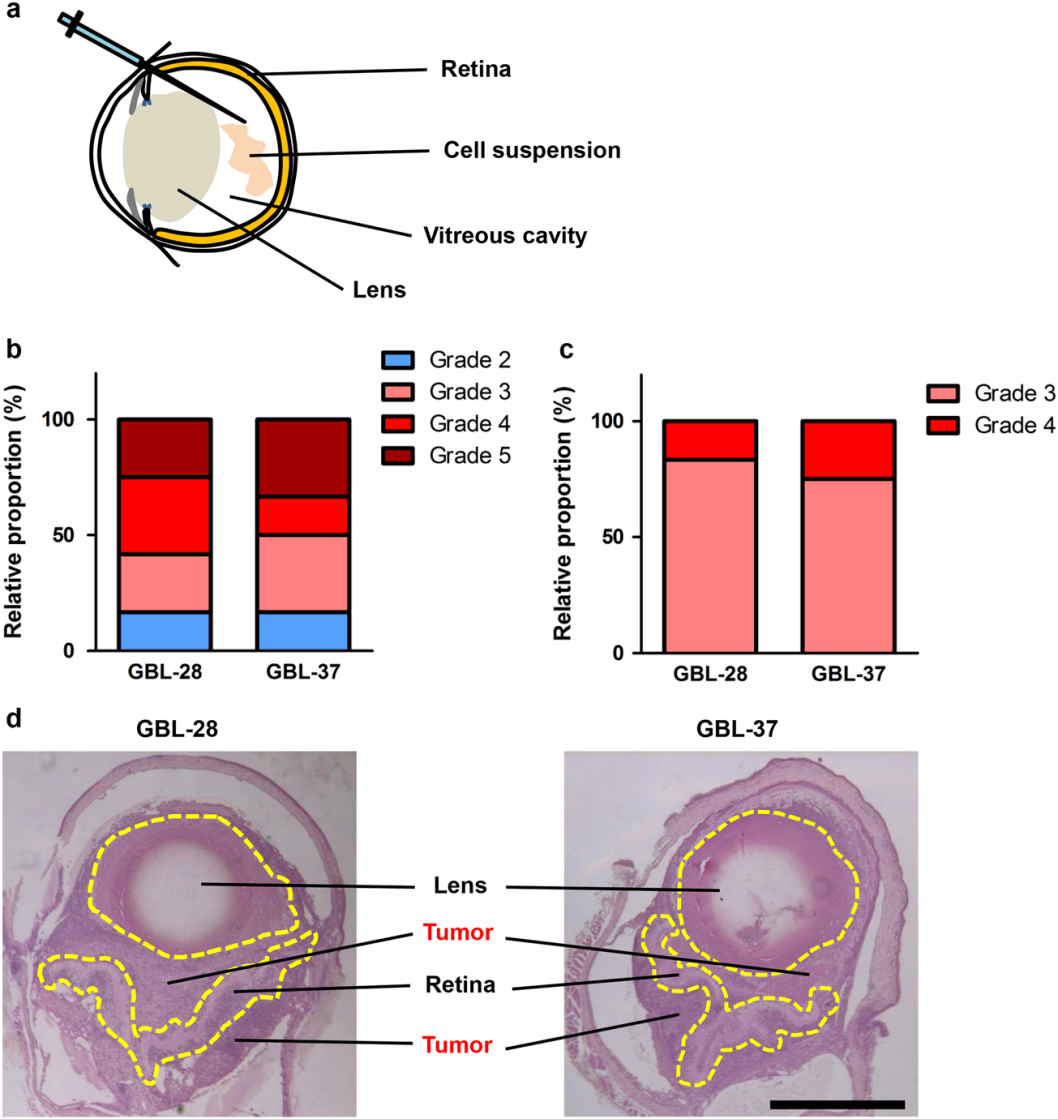
Development of a novel PDX model of glioblastoma via intravitreal injection. **a** A schematic diagram demonstrating the intravitreal injection of tumor cells. **b** The relative proportion of mice with grade 2–5 disease after the intravitreal injection of GBL-28 and GBL-37 cells that underwent normal culture conditions. **c** The relative proportion of mice with grade 3 or 4 disease after the intravitreal injection of GBL-28 and GBL-37 cells that underwent hypoxia treatment for 4 h before injection. **d** Representative images of H&E-stained sections of the eyeball at 4 weeks after the intravitreal injection of GBL-28 or GBL-37 cells that underwent hypoxia treatment for 4 h before injection. The yellow dashed lines indicate the lens and retina. Scale bar: 1 mm

**Fig. 4:**
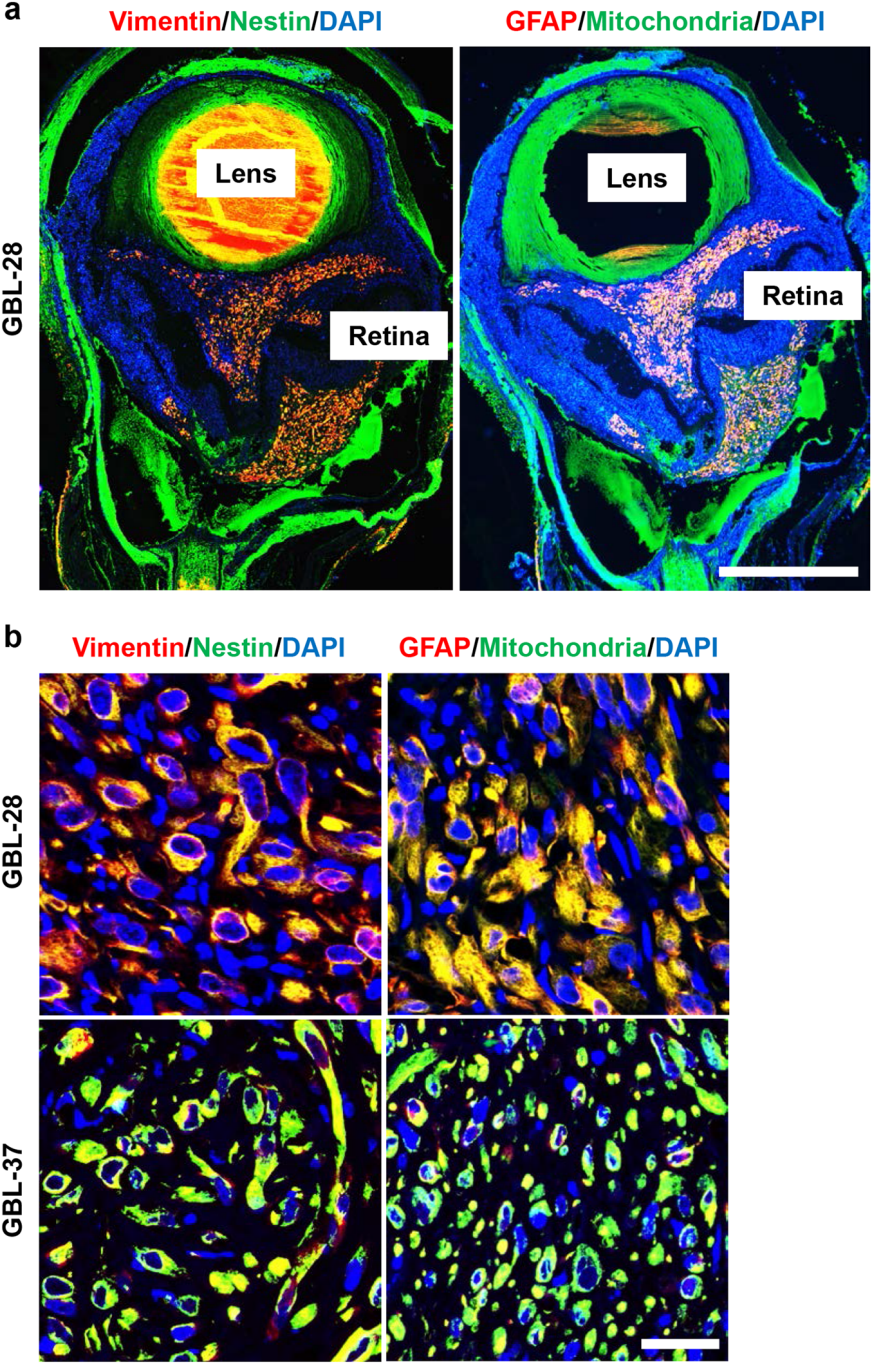
Immunohistochemical characterization of PDX tumors in the vitreous cavity. **a** Representative images of eyeball sections that were stained using DAPI and antibodies specific for human mitochondria, GFAP, vimentin, and nestin. Scale bar: 1 mm. **b** Representative magnified images of eyeball sections that were stained using antibodies specific for human mitochondria, GFAP, vimentin, and nestin. Scale bar: 20 μm

Intravitreal injection is a method used to establish orthotopic models of retinoblastoma^18^ and to deliver therapeutic agents to the retinal neuronal tissue^23^. Intravitreally administered cells primarily formed intraocular tumors in the vitreous cavity between the lens and retina (Fig. 3a). Then, the tumors could expand to the anterior chamber (between the cornea and lens) to occupy the entire eyeball. Because the vitreous cavity and retina of Balb/c nude mice can be observed with the naked eye or via an indirect ophthalmoscope, the degree of tumor formation is graded with a simple visual grading system^18^. According to the visual grading system for intraocular xenograft tumors, grades 1–5 indicate streak-like tumors, plaque-like tumors, definite mass formation, vitreous cavity-filling tumors, and eyeball enlargement, respectively^18^. Interestingly, there was a differential pattern of tumor formation between the injection of cells treated under only normal conditions (Fig. 3b) and the injection of those treated under hypoxic conditions (1% O_2_) for 4 h (Fig. 3c). The cells injected after culture under normal conditions exhibited variable tumor formation (Fig. 3b). In contrast, hypoxia treatment resulted in stable and consistent mass formation (Fig. 3c). It is also remarkable that both the GBL-28 and GBL-37 cells formed tumors extending from the vitreous cavity across the retina after hypoxia treatment (Figs. 3d and 4a), and these tumors demonstrated the invasive features of glioblastoma cells. Additional experiments using four different patient-derived cell lines also demonstrated that the injected cells effectively formed a tumor mass at 4 weeks after injection (Suppl. Figs. 1 and 2; Suppl. Tables 1 and 2).

### Immunohistochemical characterization of PDX tumors in the vitreous cavity

The original tumors from which GBL-28 and GBL-37 cells were isolated were positive for GFAP, vimentin, and nestin expression (Table 1). Based on these data, immunohistochemical characterization was performed on PDX tumors in the vitreous cavity to identify their similarity to original tumors and to confirm that they came from the injected tumor cells, not from the mouse tissue. As expected, the tumors inside the vitreous cavity and outside the retina were positive for GFAP, vimentin, and nestin expression and human mitochondria (Fig. 4a). All the markers demonstrated cytoplasmic staining patterns, indicating their intracellular locations (Fig. 4b). There were no positive signals in the uninjected eyes (Suppl. Fig. 3). In addition, the tumors stained positive for OLIG2, one of the specific markers for the glial cell lineage (Suppl. Fig. 4)^24^.

### Isolation and characterization of the tumor cells from PDX tumors in the vitreous cavity

**Fig. 5:**
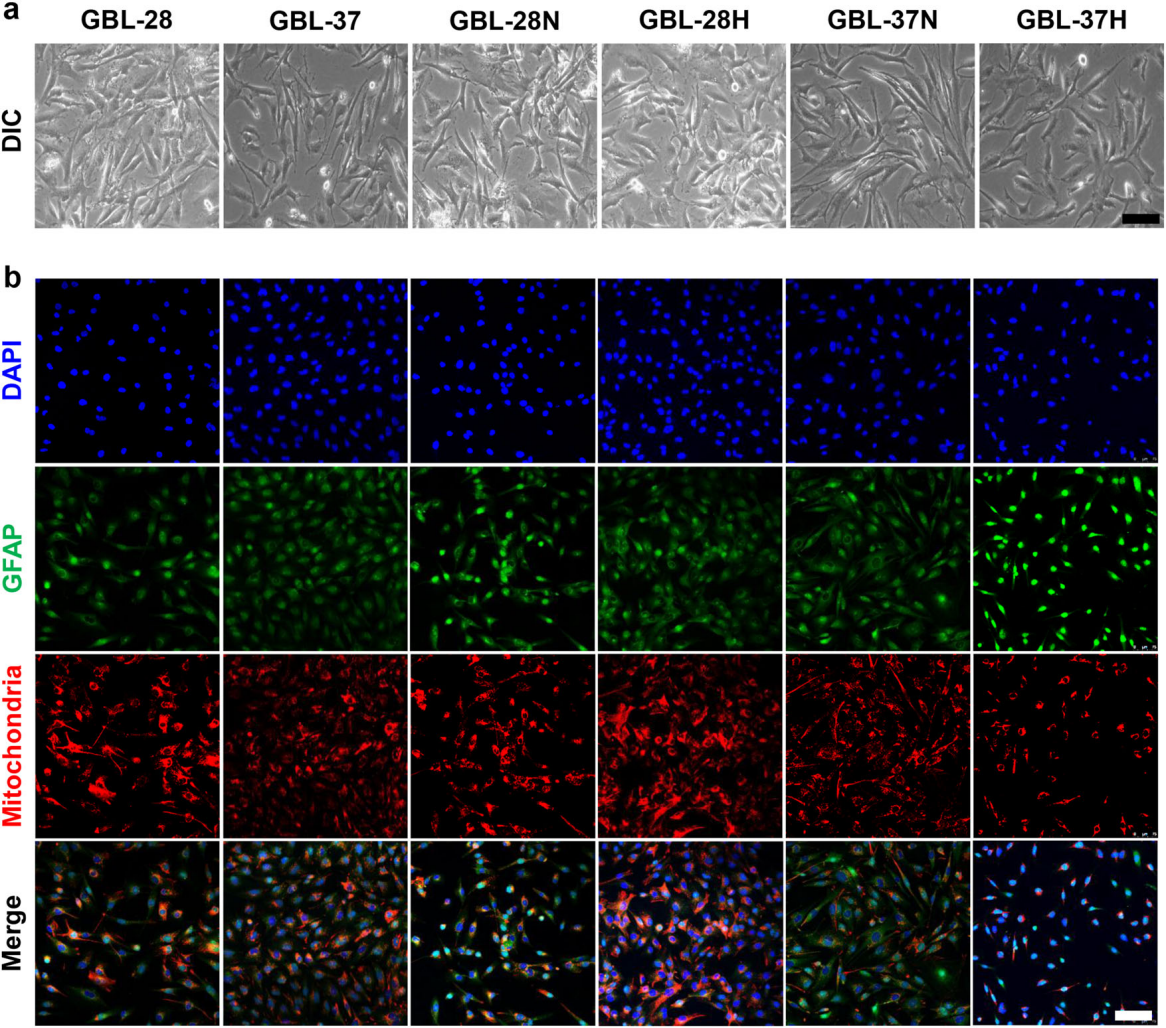
Isolation and characterization of tumor cells from the vitreous cavity of mice. **a** Representative images of glioblastoma cells. **b** Representative images of glioblastoma cells that were stained using DAPI and antibodies specific for GFAP and human mitochondria. GBL-28N and GBL-37N, cells isolated from mice at 4 weeks after the intravitreal injection of GBL-28 and GBL-37 cells that underwent normal culture conditions. GBL-28H and GBL-37H, cells isolated from mice at 4 weeks after the intravitreal injection of GBL-28 and GBL-37 cells that underwent hypoxia treatment for 4 h before injection. Scale bar: 100 μm

To further identify the characteristics of PDX tumors in the vitreous cavity, the tumors were isolated from the eyeballs and prepared for primary culture. As shown in Fig. 5a, the isolated cells retained the morphological characteristics of their parental cells. Further immunocytochemical analyses demonstrated that they exhibited similar positivity for GFAP (Fig. 5b), vimentin (Suppl. Fig. 5), and nestin (Suppl. Fig. 6).

## Discussion

In this study, a novel PDX model of glioblastoma was established via the intravitreal injection of tumor cells. It is remarkable that the patient-derived glioblastoma cells that did not form intracranial tumors at 6 weeks after injection effectively formed intraocular tumors at 4 weeks. Further experiments using four additional patient-derived cell lines showed that they also formed intraocular tumors. The confined anatomical structure might help the development of PDX tumors in vivo in the vitreous cavity. The intraocular tumors evolved from plaque-like tumors (week 2) to a certain mass (week 4), showing efficient tumor formation and development. Further immunohistochemical characterization and tumor cell isolation experiments demonstrated that the PDX tumors retained characteristics of the original tumors.

There are already established PDX models of glioblastoma generated through subcutaneous or intracranial injection^1–8^. Subcutaneously administered glioblastoma tumors are confined to the subcutaneous tissue, which is quite different from the tumor microenvironment. Furthermore, subcutaneous tumors are relatively easily accessible to therapeutic agents through systemic administration, unlike brain tumors, which are protected by the blood–brain barrier. In contrast, intracranial PDX models often take >45 days to evaluate the efficacy of therapeutic agents^6–8,25–27^, and some patients-derived cells fail to form intracranial tumors, as in this study^2^. Additionally, even though intracranial tumors develop efficiently, magnetic resonance imaging or bioluminescence imaging are required to monitor tumor formation^19,28–31^.

In this context, the PDX model established via intravitreal injection has several advantages. First, patient-derived glioblastoma cells that underwent hypoxia treatment consistently formed an intraocular mass that retained characteristics of the original tumors regarding marker expression and invasiveness within 4 weeks. Because plaque-like tumor formation was observed at 2 weeks, therapeutic options can be screened beginning at 2 weeks after injection through systemic or direct intraocular administration. Second, the retina, which is a part of the central nervous system, mimics the microenvironment of the original tumors. Stacked neuronal cells with dynamic synapses and the blood–retinal barrier system in the retinal vasculature make the retina an effective alternative to the brain. Third, compared to intracranial tumors, intraocular tumors are easily monitored through direct observation with the naked eye or an indirect ophthalmoscope with a simple optical lens (such as a 78 diopter lens). Because no additional imaging systems are required, it is easier to perform screening and monitor tumor formation. Additionally, the visual grading system provides semiquantitative scale data for quantitative analyses of tumor formation, as in this study. It is also noteworthy that hypoxia treatment, which affects the proliferation and invasion of glioblastoma cells, increased the consistency of tumor formation^32^.

The median survival of patients with glioblastoma is less than 15 months^9–11^. Therefore, an efficient and rapid screening system is required to screen second-line treatment options in patients with tumors resistant to conventional treatment. Although there have been several attempts to make a considerable reference library of glioblastoma with thorough characterization, including genome sequencing^2,33^, these efforts will be more effective with a system that can screen fresh patient-derived tumor cells more efficiently. The PDX model established via intravitreal injection is expected to screen therapeutic options within 4 weeks, which is similar to the timeframe for an orthotopically transplanted xenograft model of retinoblastoma^18,34^. Because relatively fewer cells (5–10 × 10^4^ cells) are required per mouse, several options can be evaluated simultaneously. Considering the usual preparation time for patient-derived cells (~2 weeks), all procedures can be completed in 6 weeks, which is much shorter than the median time from initial surgery to recurrence^35^. Accordingly, this model can be complementary to the intracranial model of orthotopic transplantation.

In summary, the PDX model of glioblastoma established through intravitreal injection provides an easy and efficient way to screen for tumor formation. As glioblastoma is the most common and aggressive cancer in the brain, it is imperative to develop novel therapeutic options beyond the current chemoradiation treatment. This model, which can be used 4 weeks after tumor cell injection, will be an essential tool to investigate and develop therapeutic alternatives for the treatment of glioblastoma.

## Supplementary information

Supplementary Information
