# Supplementary material for: Development of a patient-derived xenograft model of glioblastoma via intravitreal injection in mice
Source: Exp Mol Med. 2019 Apr 16;51(4):43. doi: 10.1038/s12276-019-0241-3 (PMC6467997; doi:10.1038/s12276-019-0241-3)
Supplement: Supplementary file 1 — Supplementary Information [file 12276_2019_241_MOESM1_ESM.pdf]

Supplementary Information

Development of a patient-derived xenograft model of glioblastoma via intravitreal injection in mice

Supplementary Figure 1.

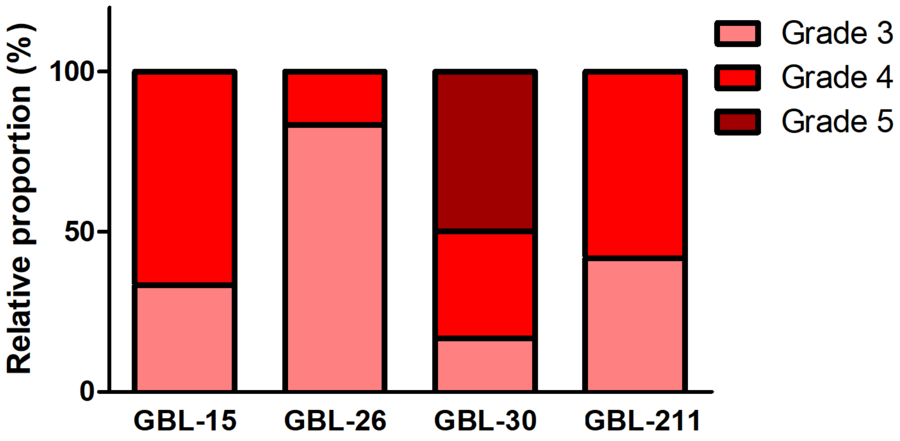

The relative proportion of mice with grade 3, 4, and 5 tumors after intravitreal injection of additional 4 patient-derived glioblastoma cell lines which underwent hypoxia treatment for 4 hours before injection.

Supplementary Figure 2.

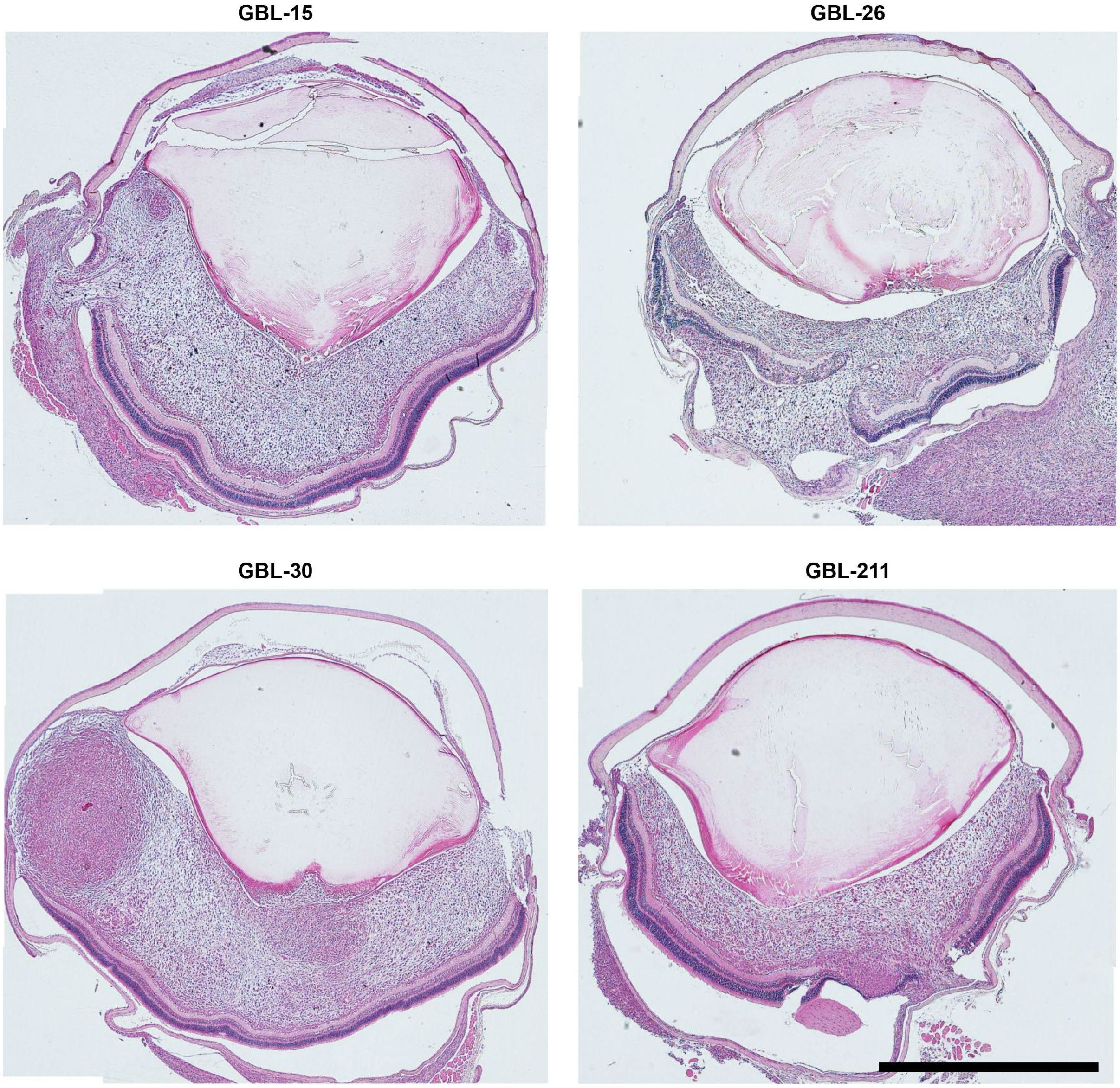

Representative photographs of H&E section of the eyeballs at 4 weeks after intravitreal injection of GBL-15, GBL-26, GBL-30, and GBL-211 which underwent hypoxia treatment for 4 hours before injection. Scale bar: 1 mm.

Supplementary Figure 3.

a b

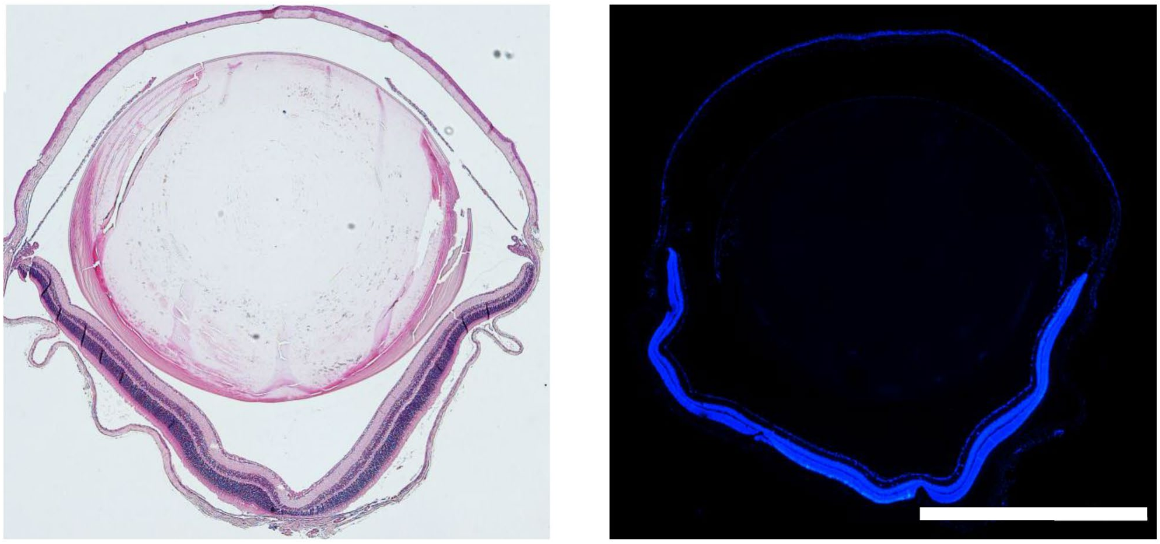

Representative photographs of H&E and immunofluorescent images of the control eyeballs of Balb/c nude mice. (a) A representative photograph of H&E sections of the control eyeballs of Balb/c nude mice. (b) A representative photograph of immunofluorescent images of the sections of the control eyeballs of Balb/c nude mice after staining with DAPI (blue) and antibodies to nestin (green) and vimentin (red). Scale bar: 1 mm.

Supplementary Figure 4.

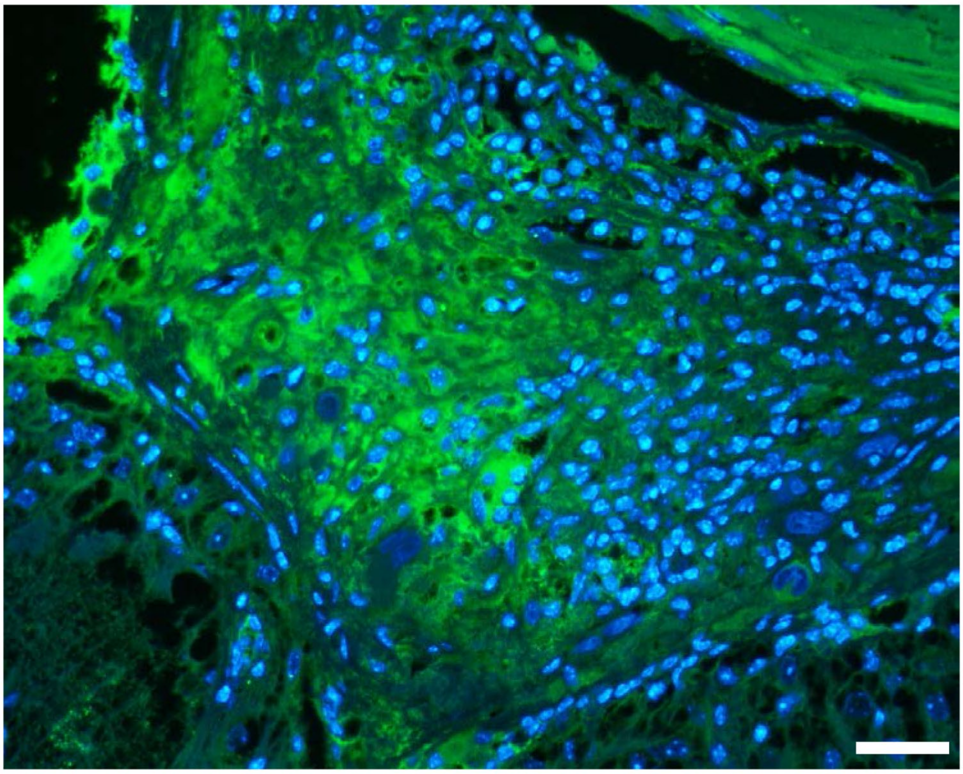

OLIG2-immunopositivity of PDX tumors in the vitreous cavity of mice. Representative magnified photographs of eyeball sections which were stained using antibodies against an antibody against human OLIG2. Scale bar: 10 μm

Supplementary Figure 5.

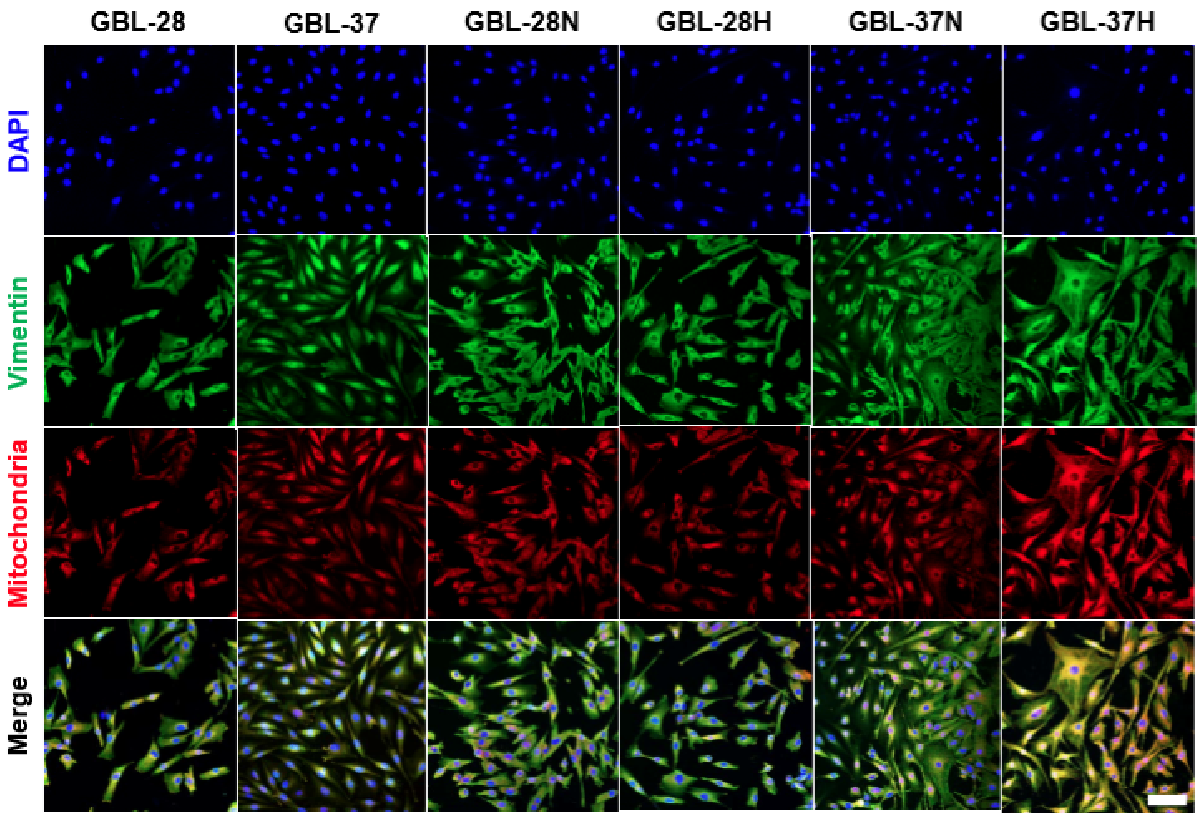

Vimentin-immunopositivity of tumor cells isolated from the vitreous cavity of mice. GBL-28N and GBL-37N, isolated from mice at 4 weeks after intravitreal injection of GBL-28 and GBL-37 cells which underwent normal culture condition. GBL-28H and GBL-37H, isolated from mice at 4 weeks after intravitreal injection of GBL-28 and GBL-37 cells which underwent hypoxia treatment for 4 hours before injection. Scale bar: 100 µm

Supplementary Figure 6.

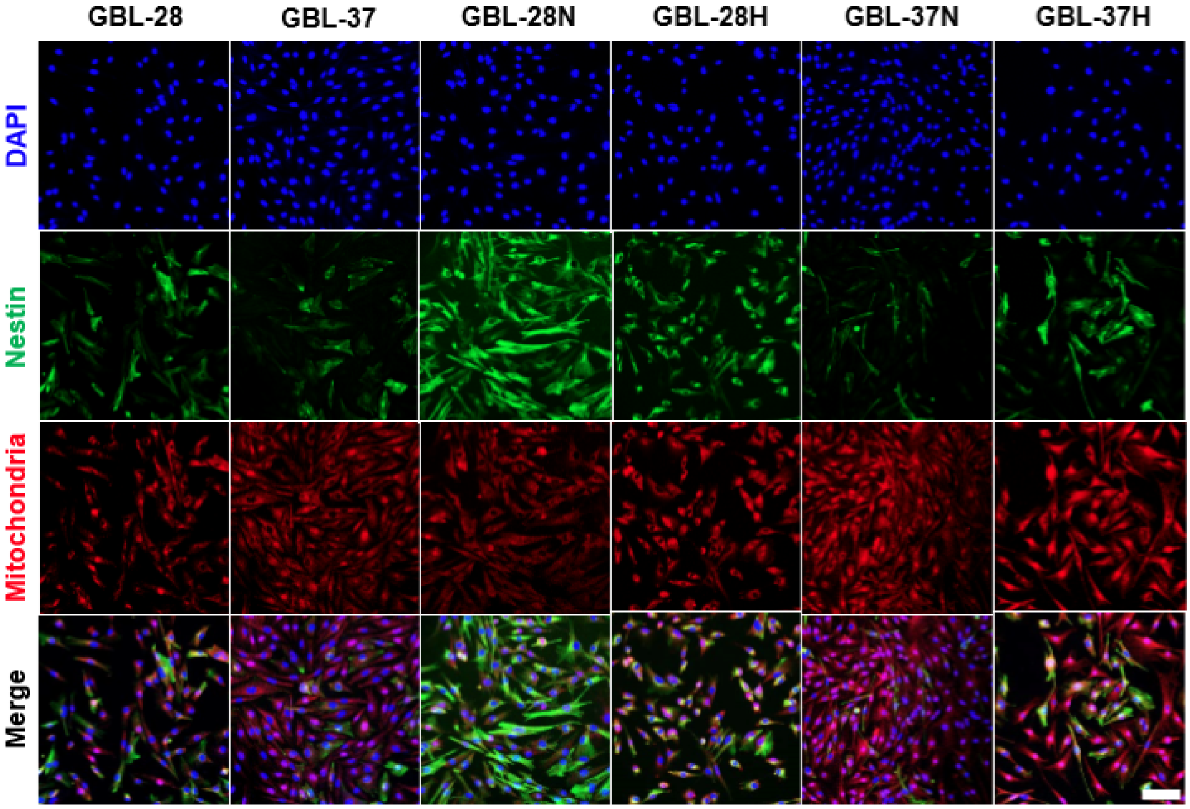

Nestin-immunopositivity of tumor cells isolated from the vitreous cavity of mice. GBL-28N and GBL-37N, isolated from mice at 4 weeks after intravitreal injection of GBL-28 and GBL-37 cells which underwent normal culture condition. GBL-28H and GBL-37H, isolated from mice at 4 weeks after intravitreal injection of GBL-28 and GBL-37 cells which underwent hypoxia treatment for 4 hours before injection. Scale bar: 100 μm

Supplementary Table 1. Demographic features of patients from whom additional glioblastoma tumors were obtained.

Features	GBL-15	GBL-26	GBL-30	GBL-211
Age at diagnosis	37	28	46	74
Sex	Female	Male	Female	Female

Supplemental Table 2. Histological and immunohistochemical characteristics of additional glioblastoma tumors.

Characteristics	GBL-15	GBL-26	GBL-30	GBL-211
WHO grade	IV/IV	IV/IV	IV/IV	IV/IV
Increased cellularity	Present	Marked	Present	Present
Nuclear polymorphism	Present	Marked	Present	Present
Mitosis (pHH3)	25/10 HPF	9/10 HPF	35/10 HPF	6/10 HPF
Vascular endothelial hyperplasia	Present	Present	Present	Present
Necrosis	Present	Present	Present	Present

HPF, high-power field; pHH3, phosphohistone H3.
